# Linear Tuning of Gamma Amplitude and Frequency to Luminance Contrast: Evidence from a Continuous Mapping Paradigm

**DOI:** 10.1371/journal.pone.0124798

**Published:** 2015-04-23

**Authors:** Gavin Perry, James M. Randle, Loes Koelewijn, Bethany C. Routley, Krish D. Singh

**Affiliations:** Cardiff University Brain Imaging Centre (CUBRIC), School of Psychology, Cardiff University, Cardiff, United Kingdom; University College London, UNITED KINGDOM

## Abstract

Individual differences in the visual gamma (30–100Hz) response and their potential as trait markers of underlying physiology (particularly related to GABAergic inhibition) have become a matter of increasing interest in recent years. There is growing evidence, however, that properties of the gamma response (e.g., its amplitude and frequency) are highly stimulus dependent, and that individual differences in the gamma response may reflect individual differences in the stimulus tuning functions of gamma oscillations. Here, we measured the tuning functions of gamma amplitude and frequency to luminance contrast in eighteen participants using MEG. We used a grating stimulus in which stimulus contrast was modulated continuously over time. We found that both gamma amplitude and frequency were linearly modulated by stimulus contrast, but that the gain of this modulation (as reflected in the linear gradient) varied across individuals. We additionally observed a stimulus-induced response in the beta frequency range (10–25Hz), but neither the amplitude nor the frequency of this response was consistently modulated by the stimulus over time. Importantly, we did not find a correlation between the gain of the gamma-band amplitude and frequency tuning functions across individuals, suggesting that these may be independent traits driven by distinct neurophysiological processes.

## Introduction

The functional role of neural oscillations in the gamma frequency bandwidth (typically 30–100 Hz) has become a topic of intense interest in recent years, particularly with respect to their role in sensory processing. MEG studies of the human visual system have shown that the frequency of the visual gamma response is variable across individuals, but stable across recording sessions within the same individual [1,2]. Visual gamma-band frequency further shows a greater degree of concordance between monozygotic than dizygotic twins[3], suggesting that it has a genetic component. The amplitude of the visual gamma response likewise appears to be variable across participants but stable across recording sessions separated over a number of weeks when analysed in source-space [2]. When analysed in sensor-space, the stability of the response is decreased, most likely due to differences in participants' head position relative to the sensors [1]. These findings would suggest that visual gamma amplitude and frequency are stable trait markers of one or more underlying physiological processes that vary across individuals.

However, while the frequency and amplitude of the visual gamma response appear to be stable across individuals for a given visual stimulus, both primate neurophysiology and human MEG studies have demonstrated that the gamma response varies systematically with changes in basic stimulus properties such as luminance contrast [4–6], size [5,7,8], orientation [9,10] and spatial frequency [11–13]. Moreover, we have recently shown that the tuning function of the gamma response varies across individuals with stimulus size, such that some individuals show a dramatic increase in gamma amplitude with increasing stimulus size while others show little difference in amplitude even with up to a twelvefold increase in size [8]. This suggests that individual differences in the properties of the visual gamma response might be due to different stimulus tuning functions of these properties across individuals. Thus, quantifying participant-level tuning functions of gamma amplitude and/or frequency might lead to further insight into the physiological basis of individual differences in the visual gamma response.

In this study we set out to determine the tuning of the visual gamma response to luminance contrast. Previous neurophysiological evidence has shown that both gamma amplitude and frequency are strongly modulated by stimulus contrast [5,6] in a broadly linear fashion [4,14], making the tuning function relatively straightforward to determine. Here, we aimed to determine the extent of individual differences in the contrast tuning functions of gamma amplitude and frequency. We further aimed to verify that these tuning functions measured non-invasively in humans using MEG were consistent with those from the primate neurophysiology literature.

Traditionally, the process of mapping the tuning curve of the gamma response to visual stimulus parameters has been achieved by using a number of discrete conditions in which stimuli are presented at a fixed parameter level in each condition. Recently, however, Ray and Maunsell [6] have presented a technique in which they modulated the contrast of a grating stimulus over time, and inferred the underlying tuning function from the corresponding modulation of the gamma response over time (in this case from intracranial recordings of macaque V1). This technique provides a potentially more efficient approach to mapping the contrast tuning function than using a number of discrete conditions with fixed parameters. Therefore, here, we mapped the tuning function of gamma amplitude and frequency to stimulus contrast using an adapted version of this approach on MEG data collected from eighteen human participants.

## Methods

### Participants

Eighteen volunteers (mean age: 22.9 yrs, range: 19–34 yrs) with normal or corrected-to-normal vision took the part in the study. Each participant gave written consent to take part in the study in accordance with The Code of Ethics of the World Medical Association (Declaration of Helsinki). All procedures were approved by the ethics committee of the School of Psychology, Cardiff University.

### Stimuli and procedure

The experimental task was based on one previously used in the 'attend centre' condition of [15], with a slight alteration to the method of joystick control. At experiment onset, the stimulus display consisted of two items on a mean luminance (26.5 cd/m^2^) grey background. A small black line (diameter 0.22°, width 0.04°) was presented at the centre of the screen, surrounded by a black circle with two gaps at opposite ends (diameter 0.35°, width 0.04°, gaps 0.07°). Participants were instructed to keep their eyes fixated on this central item at all times. The line was programmed to start rotating either clockwise or anticlockwise towards a random angle between 5 and 180° from the start orientation. The stimuli subsequently rotated towards a new random angle between 5 and 180° in the opposite direction, and so on for the duration of the experiment. The line changed 1° in orientation every 40 ms, yielding a movement that appeared visually smooth.

Participants were able to rotate the surrounding circle clockwise or anti-clockwise by pushing a MEG-compatible joystick (fORP, Cambridge Research Systems) left or right respectively, with the two gaps giving feedback about the current orientation. Participants were instructed to use the joystick with their right hand to keep the gaps in the circle aligned as closely as possible with the ends of the central line for the duration of the experiment. Under rotation, the circle rotated more quickly than the line (1° every 20 ms) so that participants could not continuously match the line's rotation but had to regularly make small adjustments to the orientation of the circle.

This task was not relevant to the main study hypothesis, but was instead intended to give a measure of task performance that could be used to indicate that the participant remained alert and fixated towards the centre of the display.

**Fig 1:**
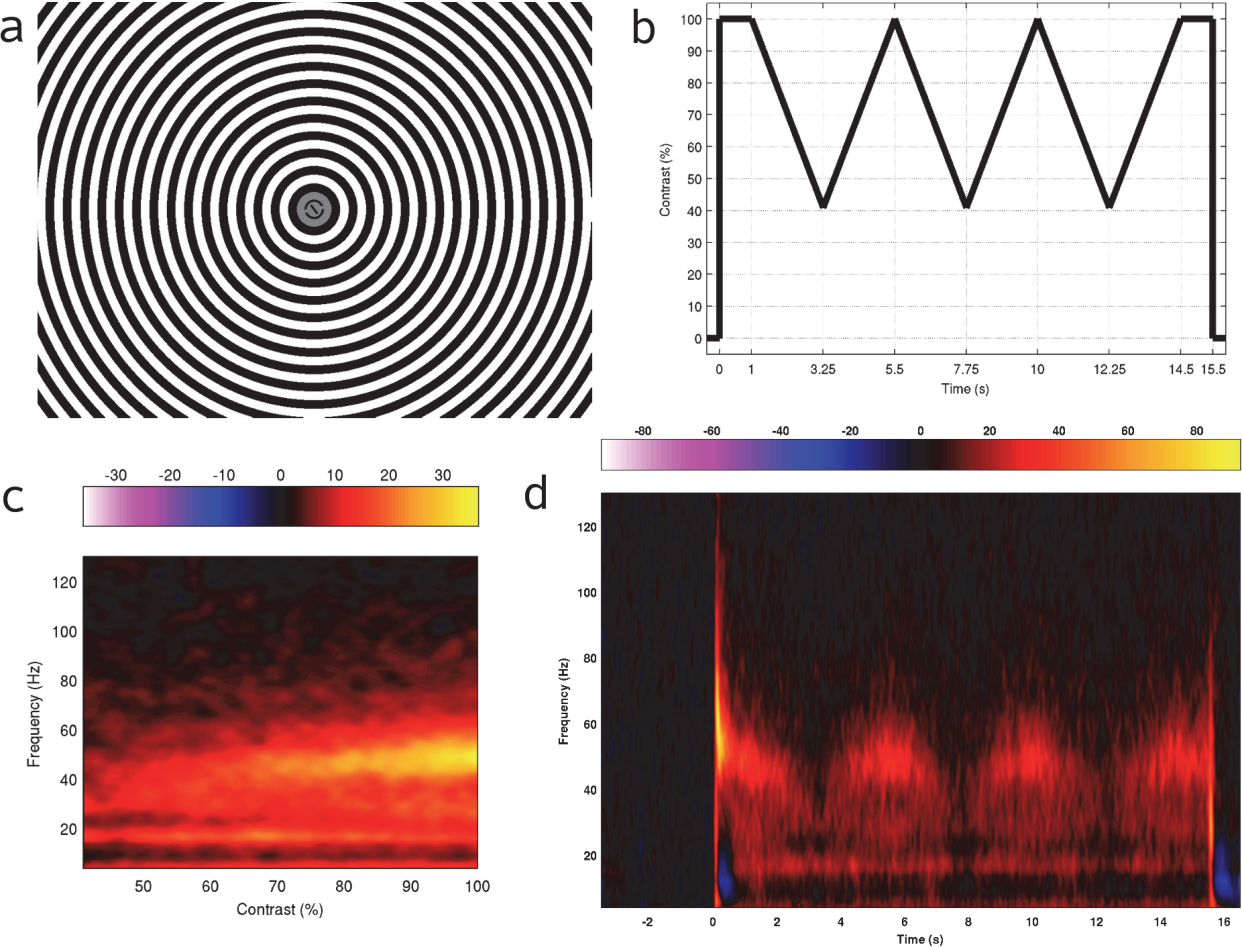
Stimulus display, temporal envelope of contrast modulation and group average spectrograms. a) Example of the visual display with stimulus at 100% contrast. b) Visual representation of the temporal envelope of contrast modulation applied during presentation of a stimulus. c) Group average spectrogram after averaging across cycles and plotting against stimulus contrast. The colour scale represents amplitude as % change from baseline. d) Group average spectrogram of the response to the visual stimulus at the location of the virtual sensors. Stimulus onset was at 0 s, stimulus modulation was from 1–14.5 s, and stimulus offset was at 15.5 s. The colour scale represents amplitude as % change from baseline.

After a random interval of length between 5–6 s, a 100% contrast, 3 cycles per degree, annular, square-wave grating was presented across the full screen (grating parameters were set based on previous evidence regarding which stimuli generate the strongest gamma response, e.g., [11,16]), with a central region of 0.67° diameter masked so that the central line and circle were still visible and participants could continue with the experimental task (see Fig 1A). 1 s after onset, the contrast began to linearly decrease in ~0.79% steps every 30 ms to 40.9% (each step corresponding to a single decrement of the 8-bit pixel intensity) before linearly increasing again back to 100% (completing a single cycle from 100% → 40.9% → 100% in 4.5 s), then decreasing and increasing again twice (thus the contrast can be viewed as having been modulated through three complete cycles of a triangular waveform of frequency 4.5 Hz). The stimulus then remained at 100% contrast for a further 1 s before offset (see Fig 1B for a visual depiction of temporal modulation of stimulus contrast over a single trial). All contrast levels used were defined by Michelson contrast and are expressed here as a percentage of the maximum displayable contrast.

We chose not to modulate the stimulus through the full range of contrasts due to previous experience that the gamma response is difficult to measure for most participants at 40% contrast (and therefore likely to be even more difficult to reliably measure at lower contrasts). Furthermore, we were keen to avoid transient responses due to the appearance of stimulus offset/onset as a result of modulations to/from 0% contrast (see Discussion).

The grating stimulus was presented 50 times throughout the task, with each presentation separated by a 5–6 s random interval. The spatial phase of the stimulus had an equal probability of being 0° or 180° on each presentation in order to prevent the build-up of retinal afterimages. After the offset of the final grating, the line and circle stimuli remained on-screen for a further 5–6 s following which the experiment was terminated.

All displays were generated by MATLAB (TheMathworks,Inc: Natick, MA) using the Psychophysics Toolbox extensions [17–19], and presented on a gamma-corrected Mitsubishi Diamond Pro 2070 monitor (1024 × 768 pixel resolution, 100Hz refresh rate).

### MEG data acquisition and analysis

Whole-head MEG recordings were made using a 275-channel CTF radial gradiometer system sampled at 1200Hz. An additional 29 reference channels were recorded for noise cancellation purposes, and the primary sensors were analysed as synthetic third-order gradiometers (Vrba and Robinson, 2001). Three of the 275 channels were turned off due to excessive sensor noise.

To achieve MRI/MEG co-registration, fiduciary markers were placed at fixed distances from three anatomical landmarks (nasion and pre-auricular) identifiable in the participants' anatomical MRIs. Fiduciary locations were verified afterwards using high-resolution digital photographs.

Data were recorded in 20 s epochs beginning at 3.5 s before grating onset. Artefact rejection was performed offline by manually inspecting the data and discarding trials with excessive muscle or head-movement-related artefacts.

After recording, each dataset was bandpass filtered using a 4^th^ order bi-directional IIR Butterworth filter at 30–70 Hz (this choice was based on the frequency range of visual gamma oscillations found across individuals in previous studies; e.g., [2]). All participants had a previous 3D FSPGR magnetic resonance imaging (MRI) scan (with 1 mm isotropic voxel resolution), acquired on a 3T GE scanner with an 8-channel receive-only head RF coil. For source localisation, a multiple local-spheres forward model [20] was derived by fitting spheres to the individual's brain surface extracted from their MRI scan using FSL's Brain Extraction Tool [21]. The synthetic aperture magnetometry (SAM) beamformer method [22] was then used to create a set of spatial filters across the whole brain at 4 mm isotropic voxel resolution for each participant. Virtual sensors were constructed from the spatially filtered data at each voxel, and from these, paired-*t* statistical images of source power (Student's t-statistic) for the period of contrast modulation (1 to 14.5 s after grating onset) contrasted with baseline (the 3 second period prior to stimulus onset) were generated for each participant.

The individual paired-*t* SAM images of each participant were examined and the coordinates of the maximum *t*-statistical value in each image was obtained (each confirmed by visual inspection to be within occipital cortex). Virtual sensor waveforms were then generated at each of the obtained voxel locations, by using the SAM beamformer method a second time, this time optimised for the single obtained (virtual) sensor location per individual. For each participant, time-frequency analysis was then performed using the Hilbert transform from 4 to 130 Hz in 0.2 Hz steps (using a bandpass 3rd order Butterworth filter with 8 Hz width). Response magnitude in the resulting spectrograms was calculated as both percentage change in amplitude relative to baseline and as absolute change from baseline. The method of calculating magnitude made little difference to our findings, so here we present only data calculated as percentage change relative to baseline. For measurement of the frequency tuning function, time-frequency analysis was repeated as above, but with 1 Hz wide filters in order to simultaneously increase both frequency resolution and temporal smoothing.

For the purposes of measuring the contrast tuning function of the gamma-band response, the time-frequency spectrograms were averaged across cycles of stimulus contrast. The first half of the averaged cycle was then reversed and averaged with the second half, in order to average out the effects of the direction of contrast change (i.e., either decreasing or increasing). This left 2.25 s of averaged time-frequency data in which contrast was a linearly increasing function of time. The contrast tuning function of amplitude was obtained by finding the maximum amplitude across frequencies in the 30–70 Hz range at each time point. The contrast tuning function of gamma frequency was achieved by fitting a Gaussian to the frequency spectrum between 20–70 Hz at each time point (20–70 Hz was chosen rather than 30–70 Hz in order to allow a Gaussian, rather than monotonic, fit when the peak frequency was close to 30 Hz—as was the case when contrast was at the lower end of the range presented). The instantaneous frequency at each time point was then defined by the peak of the Gaussian. Tuning functions could then be obtained by relating the amplitude/frequency at each time point to the visual contrast displayed at that time. As the data sampling rate (1200 Hz) was much higher than the visual contrast sampling rate (33.3 Hz), we used linear interpolation to up-sample the contrast scale. We additionally characterised the tuning functions to the beta-band response in the virtual sensors, repeating the above process but using a 10–25 Hz bandpass filter.

Confidence intervals (CIs) were estimated for gamma amplitude and frequency tuning curves using a bootstrapping procedure (as implemented by MATLAB's *bootci* function) based on 1000 resamplings of the data per amplitude or frequency measurement. Data from each half-cycle of stimulus contrast was treated as a separate sample for purposes of the bootstrap. To speed up computation time, tuning curves were down-sampled by a factor of 3 prior to calculation of CIs.

To verify that our findings were not dependent on the specific method of spectral estimation used, we replicated our visual gamma results using spectrograms generated by the Fieldtrip [23] toolbox's implementation of the multitaper method. As the results from this method closely mirrored those found using the Hilbert transform, as described in the Results section, we do not present the multitaper results in any further detail.

## Results

### Behavioural results

We calculated how well participants matched the orientation of the target line by calculating the average angular deviation of the feedback circle from the target during overlapping 10 s windows, beginning every 1 s. With one exception, this average difference was always less than 45° (the level of chance performance). For one participant, the average difference exceeded this level on some occasions, and this individual was thus excluded from further analysis. Behavioural performance in the task (as measured by average angular deviation across the whole task) did not correlate with any of the fit parameters for gamma amplitude or frequency given in the MEG results section below.

### MEG results

**Fig 2:**
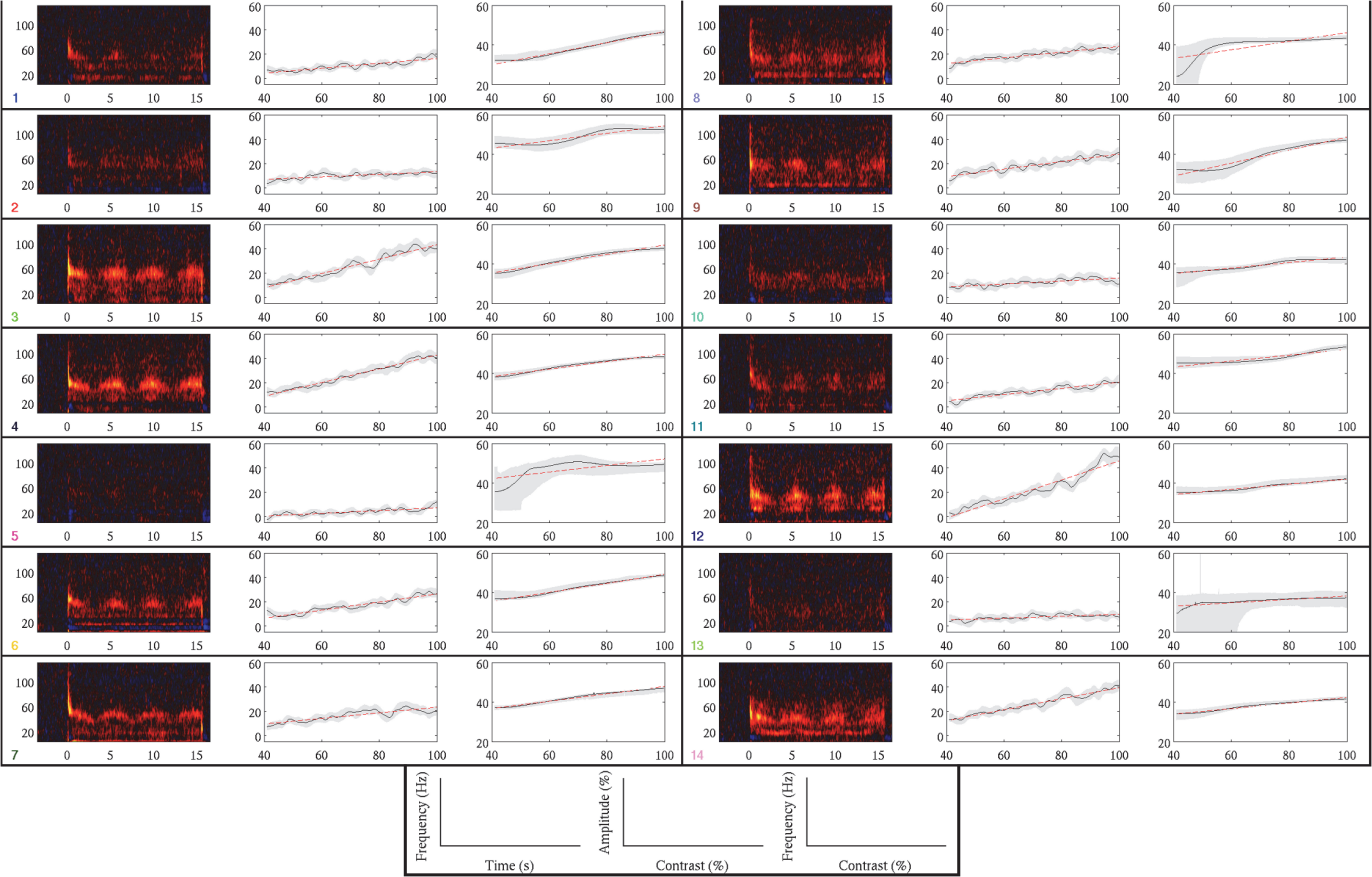
Gamma response for each participant. Each panel shows data from a single participant. Within each panel the left-hand plot shows the individual's spectrogram averaged across trials (scale ranges from +/- 150%). The middle plot shows gamma amplitude contrast (black line), 95% confidence intervals (grey shaded areas) and best fitting line (red dashed line). The right-hand plot is identical to the middle plot but for gamma frequency against contrast. The bottom panel gives axis labels for each plot The coloured number in the corner of each panel gives the participant number from Tables 2 & 3, with the colour matching the corresponding point in Fig 3.

As detailed in the Methods section, we performed a virtual sensor analysis at the peak location of visual gamma (30–70 Hz) activity to a grating stimulus in which contrast was modulated over time. These peaks always occurred within occipital cortex, in most cases close to the occipital poles (group mean Talairach coordinate of the peak: -3.3, -95.3, -3.4; see Table 1 for coordinates of individual participants). Fig 1D shows the group average spectrogram of the response to the stimulus in the virtual sensor timeseries. At stimulus onset (*t* = 0 s in the figure) we see the classic gamma-band response to visual gratings: a transient, broadband gamma 'spike' shortly after onset followed by a narrowband sustained response. During the time period that stimulus contrast was modulated (beginning at *t* = 1 s and ending at *t* = 14.5 s in the figure), there was a clear modulation of both the amplitude and frequency of the gamma response. This modulation was apparent in individual participants' spectrograms for 14 of the remaining 17 participants (see Fig 2). Three participants did not have a clear gamma response in their spectrograms and gamma frequency tuning functions (see below) could not be created for these individuals. For this reason these participants were excluded from any further analysis.

**Table 1:** Talairach coordinates of virtual sensor locations.

Participant	*x* (mm)	*y* (mm)	*z* (mm)
1	8.4	-81.3	-7.4
2	-3.8	-97.7	-2.4
3	-3.0	-107.1	-7.0
4	-24.9	-100.1	6.4
5	-18.0	-90.2	1.3
6	-5.2	-99.7	-11.3
7	11.5	-97.4	7.2
8	9.5	-95.8	-7.4
9	-11.0	-95.9	3.3
10	-13.6	-90.4	-5.7
11	-3.5	-95.0	-5.6
12	-19.7	-99.9	-6.3
13	17.4	-90.3	-10.2
14	9.1	-94.2	-2.5
Group mean (+/- se)	-3.3 (3.5)	-95.3 (1.6)	-3.4 (1.6)

For each of the 14 remaining individuals, the section of the spectrogram corresponding to the time period of contrast modulation was extracted and averaged across cycles of modulation. Then, the first half of the averaged cycle (corresponding to decreasing contrast over time) was reversed in time and averaged with the second half of the cycle (corresponding to increasing contrast over time). This had the effect of averaging together time points in the cycle at which the same visual contrast was present. We then plotted the group average of these spectrograms against contrast (Fig 1C). Again it can be seen that both gamma amplitude and frequency were modulated by stimulus contrast, and it becomes apparent from these plots that, in the group average at least, this modulation takes the form of increasing amplitude and frequency with increasing contrast.

To fit the contrast tuning function of amplitude for each participant, we found the mean amplitude across frequency (within the 30–70 Hz range) at each time point. Consistent with previous MEG studies [14,24], the relationship between contrast and amplitude was monotonically increasing and appeared to be broadly linear, so we performed linear fits of these data (see Table 2 for fit parameters and confidence intervals, and Fig 2 for plots by participant of amplitude against contrast along with lines of best fit). In all cases fit gradients were positive (median = 0.24), which was significantly different from chance (*p* = 1.2e-4; sign test). This indicated that there was a consistently positive relationship between stimulus contrast and gamma amplitude. However, the magnitude of the fit gradient varied substantially between participants (by approximately a single order of magnitude). We found that gamma amplitude at 100% contrast was highly correlated with the linear fit gradient (*r* = 0.98, *p*< 1e-7 based on a permutation test), suggesting that the difference in gamma amplitude between participants was largely explained by differences in the contrast gain of the gamma response.

**Table 2:** Linear fit parameters of gamma amplitude and frequency tuning to contrast.

Particip.	Amplitude		Frequency	
	Gradient [95% CI]	y-intercept	Gradient	y-intercept
1	0.201 [0.195, 0.207]	-3.84 [-4.29, -3.39]	0.280 [0.278, 0.282]	18.66 [18.51, 18.80]
2	0.103 [0.098, 0.108]	2.80 [2.43, 3.18]	0.188 [0.183, 0.193]	35.60 [35.24, 35.95]
3	0.590 [0.580, 0.599]	-15.81 [-16.48, -15.14]	0.235 [0.232, 0.238]	25.98 [25.79, 26.17]
4	0.566 [0.561, 0.571]	-14.01 [-14.39, -13.63]	0.190 [0.188, 0.192]	30.76 [30.61, 30.91]
5	0.114 [0.109, 0.119]	-4.32 [-4.67, -3.96]	0.167 [0.155, 0.178]	35.48 [34.63, 36.33]
6	0.331 [0.324, 0.338]	-6.91 [-7.39, -6.42]	0.227 [0.226, 0.228]	26.43 [26.33, 26.53]
7	0.229 [0.221, 0.237]	0.23 [-0.36, 0.83]	0.186 [0.185, 0.188]	29.21 [29.09, 29.32]
8	0.232 [0.227, 0.237]	2.85 [2.48, 3.22]	0.217 [0.206, 0.229]	24.40 [23.56, 25.24]
9	0.309 [0.302, 0.315]	-3.17 [-3.61, -2.72]	0.328 [0.323, 0.332]	15.89 [15.54, 16.23]
10	0.123 [0.117, 0.129]	3.49 [3.06, 3.93]	0.140 [0.138, 0.142]	29.47 [29.31, 29.62]
11	0.255 [0.248, 0.261]	-5.21 [-5.69, -4.74]	0.150 [0.146, 0.153]	37.24 [36.99, 37.49]
12	0.782 [0.766, 0.796]	-32.57 [-33.64, -31.50]	0.131 [0.129, 0.132]	28.92 [28.82, 29.01]
13	0.082 [0.077, 0.087]	1.39 [1.04, 1.75]	0.086 [0.083, 0.089]	29.57 [29.36, 29.78]
14	0.454 [0.448, 0.459]	-6.14 [-6.53, -5.75]	0.142 [0.140, 0.143]	28.15 [28.04, 28.26]

To map the contrast tuning function of frequency, we took the averaged spectrograms and at each time point performed a non-linear fit of the frequency spectrum using a Gaussian function. The instantaneous frequency at each time point was then defined as the peak of the Gaussian. We initially performed this fitting process only on the spectrum from 30–70 Hz. However, at low contrasts the peak frequency occurred close to the lower edge of this bandwidth, which affected the quality of the Gaussian fit. Therefore, we re-ran the analysis using a 20–70 Hz bandwidth, and found that this improved the fits. When performed on the initial spectrograms, these fits were also highly susceptible to temporal noise (particularly at low contrast values were the amplitude of the gamma response was weak). We therefore regenerated the spectrograms with the frequency bandwidth at each frequency point reduced from 8 Hz to 1 Hz in order to increase temporal smoothing (with the added benefit that this also increased frequency resolution).

As with the contrast tuning function of gamma amplitude, the tuning function of gamma frequency appeared to be broadly linear (Fig 2). We again performed linear fits of the tuning functions (see Table 2 for fit parameters and statistics). Similar to amplitude, the frequency fit gradients were positive in all cases (median = 0.19), indicating that there was a positive relationship between visual contrast and gamma frequency. However, the inter-participant variance of the gradient of the frequency tuning was less than that found for amplitude (by approximately an order of magnitude), suggesting that the contrast tuning function was more consistent across participants for frequency than amplitude. Moreover, we did not find a strong correlation between the gamma frequency at 100% contrast and the fit gradient (*r* = 0.39, *p* = 0.17 based on a permutation test), in contrast to our findings for gamma amplitude. Taken together, these findings suggest that individual differences in the contrast gain function of frequency may be relatively small, implying that contrast gain functions play only a minor part in individual differences in gamma frequency.

**Fig 3:**
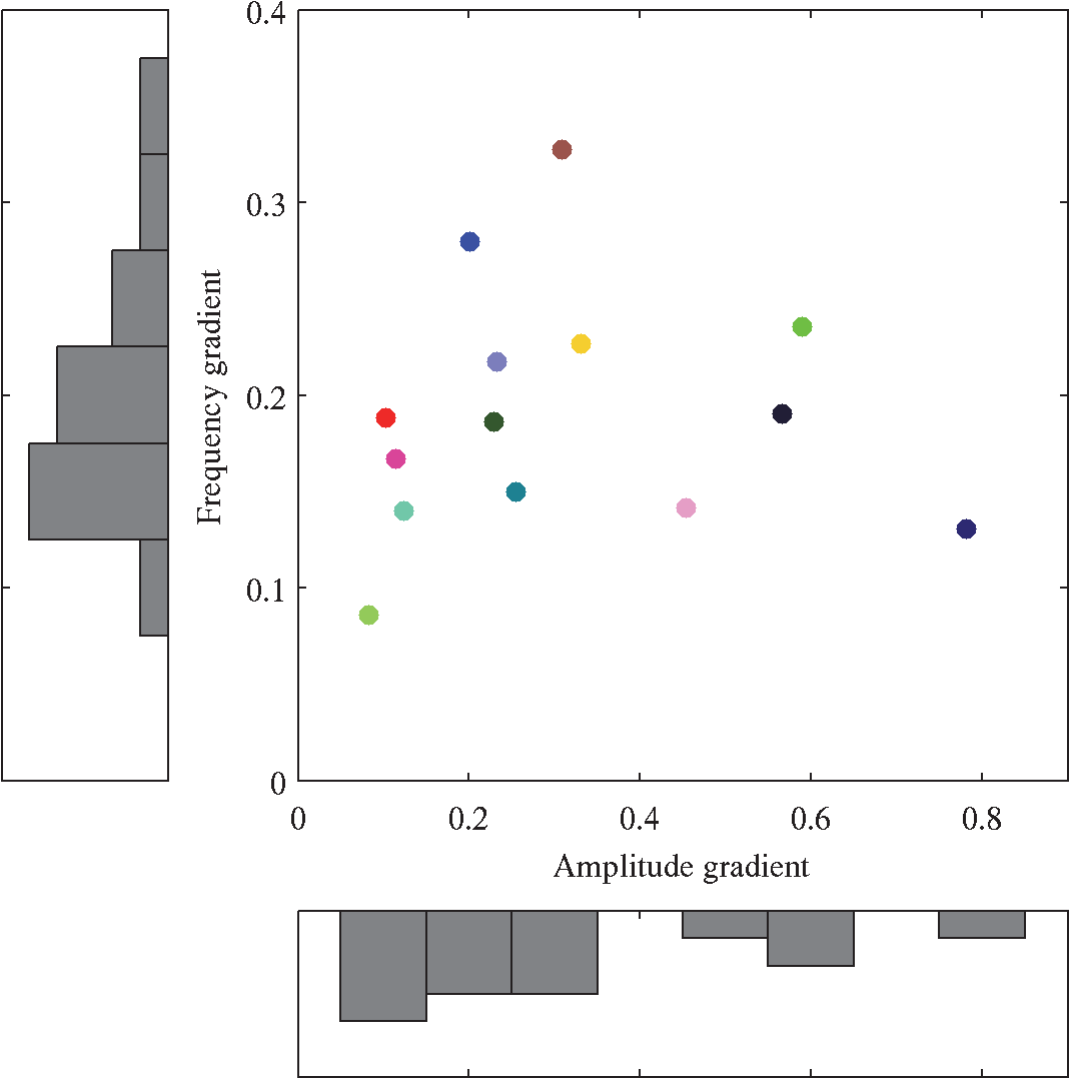
Comparison of fit gradients for gamma amplitude and frequency. Plot showing the linear fit gradient of the frequency tuning function against the linear fit gradient of the amplitude tuning function for each participant, alongside histograms showing the group distribution of each parameter. The colour of each point in the central plot matches the colour of the lines of the corresponding participant number in Fig 2.

A further distinction between the role contrast tuning plays in determining gamma amplitude and frequency was that we did not find any correlation between the gradients of the fits to amplitude and frequency across participants (*r* = 0.04, *p* = 0.87 based on a permutation test; see Fig 3). This suggests that the two functions may be driven by different underlying factors.

Fig 1C also reveals the existence of increased power in the beta-band, centred around 17 Hz. We had not anticipated the presence of this beta response and were interested to determine whether, as with the gamma response, it was also modulated by stimulus contrast. The median gradient of the linear fits was 0.053 (Table 3) which was not significantly different from zero (*p* = 0.79; sign test). Thus, beta-band amplitude did not show a clear relationship to stimulus contrast.

**Table 3:** Linear fits of beta amplitude and frequency tuning to contrast.

Particip.	Amplitude		Frequency	
	Gradient [95% CI]	y-intercept	Gradient (x100)	y-intercept
1	0.128 [0.121, 0.136]	3.07 [2.51, 3.62]	1.942 [1.869, 2.016]	12.67 [12.62, 12.72]
2	-0.040 [-0.049, -0.03]	2.94 [2.29, 3.58]	N/A	N/A
3	0.328 [0.317, 0.338]	-7.11 [-7.85, -6.37]	N/A	N/A
4	0.058 [0.049, 0.068]	2.60 [1.90, 3.30]	N/A	N/A
5	-0.005 [-0.012, 0.002]	0.50 [0.10, 0.90]	N/A	N/A
6	-0.116 [-0.128, -0.104]	12.39 [11.52, 13.27]	0.497 [0.467, 0.525]	14.15 [14.13, 14.17]
7	0.177 [0.168, 0.185]	4.68 [4.04, 5.31]	1.810 [1.725, 1.896]	16.31 [16.24, 16.37]
8	0.148 [0.136, 0.160]	7.16 [6.30, 8.03]	1.077 [1.018, 1.137]	16.77 [16.72, 16.81]
9	-0.023 [-0.041, -0.005]	15.98 [14.67, 17.30]	1.352 [1.288, 1.417]	15.89 [15.54, 16.23]
10	-0.024 [-0.031, -0.017]	3.13 [2.61, 3.66]	N/A	N/A
11	0.163 [0.154, 0.171]	-2.23 [-2.85, -1.61]	-0.457 [-0.473, -0.441]	14.75 [14.74, 14.76]
12	0.198 [0.186, 0.211]	-5.46 [-6.35, -4.56]	N/A	N/A
13	0.048 [0.039, 0.057]	2.47 [1.80, 3.13]	-1.540 [-1.740, -1.340]	15.77 [15.63, 15.92]
14	-0.111 [-0.121, -0102]	33.12 [32.44, 33.82]	6.438 [6.381, 6.495]	13.11 [13.07, 13.15]

We further characterised the beta-band frequency response using the Gaussian fitting method outlined above. Seven participants had spectral fits that were within the 10–25 Hz bandwidth for all contrasts, and a further participant had spectral fits when a slightly shifted bandwidth of 8–24 Hz was used. The remaining six participants were excluded from further analysis. We again performed linear fits of the frequency response (Table 3). While one individual (participant 14) did appear to show a beta frequency that consistently increased with stimulus contrast, the remaining participants demonstrated only a moderate relationship between frequency and contrast at best. Thus, the median fit gradient was only 0.013, which was not significantly different from zero (*p* = 0.29; sign test).

## Discussion

In this experiment we used a method of continuously modulating luminance contrast over time in order to map individual differences in the contrast tuning functions of both the amplitude and frequency of the visual gamma response. We found that, in general, both amplitude and frequency were linearly-increasing with stimulus contrast, but that there was substantial inter-individual variability in the gain (as reflected in the linear gradient) of the tuning functions. Importantly, individual contrast tuning functions of amplitude and frequency did not correlate, suggesting that these two measures of oscillatory neural activity may be driven by separate underlying parameters.

We additionally observed the presence of stimulus-induced beta-band activity in the group average spectrogram. However, we did not find any evidence for a consistent modulation across individuals of either the amplitude or frequency of this response with respect to contrast. Thus, the beta-band response to visual stimulation appears to be sensitive to the presence of the stimulus, but not to its contrast.

To our knowledge, the current study is the first in the MEG literature to use this method of continuously modulating the stimulus parameter of interest in order to map the tuning curve of an oscillatory response over time. The methodology was inspired by a previous study conducted in primates by Ray & Maunsell [6] in which the gamma response was measured in V1 while the contrast of grating stimuli was temporally modulated. One difference between our study and this previous work is that they modulated contrast over the full displayable range (from 0% to 100%) whereas we used a restricted range of mid to high contrasts (from 40.9% to 100%). Our reasons for this were twofold. Firstly, based on previous work [14] we considered it unlikely that we would be able to reliably measure gamma at contrasts much below 40% using MEG (likely due to the poorer signal-to-noise ratio achievable extracranially than can be achieved from invasive recordings). Secondly, modulating the stimulus away from or towards 0% contrast would induce transient responses due to apparent stimulus offset and onset, and these responses could contaminate our measures of gamma amplitude and frequency. The presence of such onset and offset effects likely explains why the modulation of the gamma response appeared asymmetric with regards to increasing and decreasing contrast in the Ray and Maunsell study, but not in the present study. In this sense, we consider our methodology to have been more suitable for accurately measuring the contrast tuning function of gamma over the range of contrasts tested, but at the expense of being unable to measure the tuning function at lower contrasts.

A linear relationship between gamma frequency and stimulus contrast has previously been found in intracranial recordings in primates [5,6], suggesting that our data is in good agreement with the underlying neurophysiology. The relationship between our gamma amplitude findings and the primate literature is more complex, however. While early studies found gamma amplitude to be monotonically increasing with contrast [25,26], more recent work has suggested that gamma amplitude may saturate, or even decrease, at high contrasts [5,6] (and in at least one study the relationship appeared to be different for different monkeys [27]). We note however, that the different spatial scale of the measurements (within a small region of perhaps 500μm for electrode recordings [28], versus several centimetres for MEG) means that there are scenarios for which a saturating local contrast response function could lead to an increasing response function when measured extracranially with MEG. For instance if, at higher contrasts, the spatial coherence—but not the power—of the gamma signal increased, this would be manifested as a saturation of the gamma amplitude in electrode recordings, but an increase in amplitude in MEG. Alternatively, changes in the spatial distribution of gamma sources at high contrasts might not easily be detectable in intracranial local field potential (LFP) recordings without specifically mapping signal across the cortical surface of V1, but could lead to differences in the tuning functions measured intracranially and extracranially. Thus, only when the precise relationship between gamma amplitude and luminance contrast can be determined unambiguously in primates can we begin to relate our results to the underlying neurophysiological response function.

In the absence of a 'ground truth' from LFP recordings we can still have some confidence of the validity of the amplitude tuning functions found here. This is because previous work found a linear relationship between gamma amplitude and stimulus contrast in humans using MEG [4] (see also [14]). In that study, however, data were presented only in the form of the group-averaged normalised gamma amplitude, and information about individual differences in contrast gain of the gamma response was not reported. Thus, the current study is the first to demonstrate individual differences in contrast gain of the amplitude of the visual gamma response, as well as the first to measure the contrast-frequency relationship of visual gamma oscillations in humans.

It is notable that the linear tuning function found here for gamma amplitude differs greatly to the contrast tuning functions of neural firing rates, which tend to be highly non-linear and are better characterised by a hyperbolic ratio [29]. This lends further weight to the previously found dissociation between firing rates and the narrow-band gamma response [7,30,31] and is evidence that, unlike broadband/high-frequency gamma, narrow-band gamma is not directly coupled to local spiking activity but is instead a distinct phenomenon.

Previous MEG studies of the visual gamma response using a single stimulus contrast have found that the frequency and amplitude of the response varies substantially across individuals [1,2]. Our data suggest that individual differences in gamma amplitude to a stimulus at 100% contrast are largely due to individual differences in the contrast gain of the amplitude. Similarly, we found that differences in the contrast gain of gamma frequency partially explained individual differences in the gamma frequency induced by a 100% contrast stimulus. However, this was a relatively small effect and gain explained frequency to a much lesser extent than was the case for gamma amplitude.

One caveat, however, is that we cannot completely exclude the possibility that differences in measured amplitude between participants are confounded by the effects of the MEG lead field, and particularly by differences in source orientation between participants. Because radially-oriented sources do not generate an external magnetic field in spherical volume conductors, we would expect sources to produce a greater signal projection on to the MEG sensors as their orientation moves away from the radial axis. For this reason, if the angle of the gamma source varied substantially across participants relative to the scalp surface we would also expect substantial variance in the slope of the tuning function, even if the true slope did not differ. However, because our stimulus was large (10.7° x 8°) and would have stimulated an extended region of visual cortex, we consider it likely that the gamma response was generated by sources at a mixture of orientations, which would lessen or even eliminate this potential effect. Thus, although it cannot be determined with absolute certainty without more detailed studies in which the true source distribution is determined independently, we would suggest that within-participant differences in contrast gain of the gamma amplitude found here are due primarily to true physiological differences, rather than the effects of the MEG lead field.

Evidence from both empirical and modelling studies indicates that cortical oscillations in the gamma frequency range are due to the generation of so-called 'pyramidal-interneuron network gamma' (PING) oscillations within local circuitry [32–36]. The PING model suggests that under tonic drive (received either directly through monosynaptic input or disynaptically via pyramidal cells) fast-spiking inhibitory interneurons produce synchronised discharges at the gamma frequency. These discharges, in turn, generate oscillatory post-synaptic potentials in the principal pyramidal cells. These post-synaptic potentials manifest at a population level in the electroencephalogram or magnetoencephalogram as the gamma response. Thus, it has been suggested that individual differences in properties of the gamma response may directly reflect inter-participant variability in the neurophysiological parameters of the PING mechanism, such as the strength of GABAergic inhibition [37]. Our data provide further elaboration to this view by suggesting that inter-participant differences are, at least in part, due to differences in the contrast gain of gamma amplitude (and, to a lesser extent, frequency). Therefore, we suggest that future work aimed at uncovering which parameters of the PING mechanism determine the amplitude and frequency of gamma should focus on those parameters that are also involved in determining the contrast gain response of visual gamma. Recent modelling has begun to identify some of the parameters which are critical in determining the contrast gain of gamma [38].

Interestingly, we did not find evidence for a correlation between the contrast gain of gamma amplitude and gamma frequency across participants. It is possible that this could be a false negative finding due to the unreliability of the linear fits in those participants with a relatively weak gamma response to visual stimulation. However, even if we excluded participants with the weakest gamma response (and hence those with the smallest gradients—i.e., those to the far left of Fig 3) there would still be no positive relationship between the remaining data points. Thus, our data suggest that the gain of the amplitude and frequency tuning functions are independent of each other and that they may be determined by different neurophysiological parameters. Interestingly, recent modelling work has suggested that disynaptic (reflecting afferent drive to local pyramidal cells) and monosynaptic inputs (reflecting recurrent drive from lateral and/or top-down connections) to inhibitory interneurons modulate gamma amplitude and frequency differently [39]. This suggests that the balance of these different connection pathways may play a role in determining the different amplitude and frequency tuning functions.

In conclusion, the methods presented here have enabled us to determine the presence of a linear tuning function for both gamma amplitude and frequency in occipital cortex to luminance contrast in a group of fourteen neurotypical participants. We have also been able to demonstrate substantial inter-individual variability in the slope of these tuning functions (particularly for amplitude). Importantly, the contrast gain of gamma amplitude and frequency did not correlate with each other. Further investigation into the underlying causes of this variability could provide important evidence for the relationship between measurable parameters of the visual gamma response and properties of the neuronal networks involved in generating the gamma response.
